# CBMAT: a MATLAB toolbox for data preparation and post hoc analyses in neuroimaging meta-analyses

**DOI:** 10.3758/s13428-023-02185-3

**Published:** 2023-08-01

**Authors:** Jordi Manuello, Donato Liloia, Annachiara Crocetta, Franco Cauda, Tommaso Costa

**Affiliations:** 1 GCS-fMRI, Koelliker Hospital and Department of Psychology, University of Turin, Turin, Italy; 2 FOCUS Lab, Department of Psychology, University of Turin, Turin, Italy; 3 Move’N’Brains Lab, Department of Psychology, University of Turin, Turin, Italy; 4 Neuroscience Institute of Turin (NIT), Turin, Italy

**Keywords:** Coordinate-based meta-analyses, MATLAB, Data preparation, Validation analyses, Activation likelihood estimation, BrainMap, Quantitative synthesis

## Abstract

Coordinate-based meta-analysis (CBMA) is a powerful technique in the field of human brain imaging research. Due to its intense usage, several procedures for data preparation and post hoc analyses have been proposed so far. However, these steps are often performed manually by the researcher, and are therefore potentially prone to error and time-consuming. We hence developed the Coordinate-Based Meta-Analyses Toolbox (CBMAT) to provide a suite of user-friendly and automated MATLAB® functions allowing one to perform all these procedures in a fast, reproducible and reliable way. Besides the description of the code, in the present paper we also provide an annotated example of using CBMAT on a dataset including 34 experiments. CBMAT can therefore substantially improve the way data are handled when performing CBMAs. The code can be downloaded from https://github.com/Jordi-Manuello/CBMAT.git.

## Introduction

Coordinate-based meta-analyses (CBMAs) are a useful and widely implemented quantitative tool in the field of human neuroimaging research (Salimi-Khorshidi et al., 2009). Their ultimate goal is to measure consensus between results from multiple peer-reviewed experiments that used structural or functional neuroimaging techniques (Caspers et al., 2014). Unlike image-based meta-analyses that take as input the complete 3-D maps of the results obtained in each experiment, the starting point for CBMAs is the list of the stereotactic coordinates (x,y,z) locating the observed peaks of effect (technically named foci).

Different algorithms have been developed to then reconstruct the 3-D maps from the foci (Radua & Mataix-Cols, 2012; Wager et al., 2007). In the present work, we mainly refer to activation likelihood estimation (ALE) (Eickhoff et al., 2009; Eickhoff et al., 2012; Turkeltaub et al., 2012), currently the most widely used approach in the field of CBMA (Tahmasian et al., 2019). According to the BrainMap archive (http://brainmap.org/pubs/), this algorithm has been used in more than 1100 publications in the last 20 years, investigating healthy and pathological populations on the basis of both functional and structural neuroimaging data. Briefly, this procedure consists of two main steps. First, for each experiment included in the dataset, a modeled activation (MA) map is created, modeling each focus using a 3D Gaussian density distribution with a specified variance accounting for the number of subjects included in that specific experiment (Eickhoff et al., 2009). Secondly, the final ALE map is obtained from the union of all the MA maps. A range of techniques is then available to assess the statistical significance threshold, the description of which is beyond the scope of this work.

While the computation of the consensus estimate is the core of each CBMA, several procedures have to be implemented before and after it. Crucially, these further steps often require the manual intervention of the researcher, and are therefore potentially prone to error which could lead to biased findings (Manuello, Costa, et al., 2022a). When performing a CBMA, most of the time needed is spent building the dataset. In fact, this kind of analysis benefits from the collection of the greatest possible pool of experiments, so as to mitigate the effect of potential outliers or spurious results (Fox et al., 2014). In doing this, strict inclusion criteria must be followed (Müller et al., 2018; Page et al., 2021; Tahmasian et al., 2019). While CBMAs provide an informative output per se, allowing one to overcome the variability in results often found among experiments (Bowring et al., 2019; Li et al., 2020; Smith et al., 2005), a range of post hoc analyses have been proposed in recent years. They are intended to provide further validation for the evidence found through the main CBMA, and include leave-one-experiment-out (LOEO), split-half analysis and subset analysis. To the best of our knowledge, the only resource currently available to improve at least part of these processes is the Python tool NiMARE (Neuroimaging Meta-Analysis Research Environment) (https://nimare.readthedocs.io/en/latest/index.html - ). Although Python is a widely used environment, some researchers may prefer to operate with a different language. The aim of the Coordinate-Based Meta-Analyses Toolbox (CBMAT) is therefore to provide a MATLAB® suite of user-friendly and automated functions allowing one to prepare data for the main CBMA analysis and post hoc analyses in a fast, reproducible and reliable way.

CBMAT was coded in MATLAB® R2016a, and consists of seven functions:

A) **load_foci**: Imports foci into MATLAB®
B) **remove_multiple**: Removes multiple experiments included in the same paper
C) **filter_by_tissue**: Selects foci in gray matter- or white matter-only templates
D) **prepare_MACM**: Prepares data for meta-analytic connectivity modeling
E) **create_LOEO**: Prepares data for leave-one-experiment-out analysis
F) **create_subsets**: Prepares data for split-half or subset analysis
G) **dataset_hist**: Creates histograms for several features of the dataset

Notably, removal of multiple experiments from the same paper and creation of histograms are not implemented in NiMARE. The typical workflow of CBMAT is to import data with **load_foci**, prepare data for the main CBMA with **remove_multiple** and/or **filter_by_tissue** and/or prepare_**MACM**, prepare data for post hoc analyses with **create_LOEO** and/or **create_subsets**. At any stage, **dataset_hist** can be used to inspect the obtained dataset. The output of each function (excluding **dataset_hist**) is a .txt file ready to be analyzed with third-party software. CBMAT is optimized for compatibility with Sleuth (http://brainmap.org/sleuth/) (Fox et al., 2005) and GingerALE (http://brainmap.org/ale/) (Eickhoff et al., 2012), both freeware tools developed by BrainMap (Laird et al., 2009). The same algorithm invoked through GingerALE’s interface can be run from command line (http://brainmap.org/ale/cli.html) and directly included in a MATLAB® code. **filter_by_tissue** and **prepare**_**MACM** require **Tools for NIfTI and ANALYZE image** (https://www.mathworks.com/matlabcentral/fileexchange/8797-tools-for-nifti-and-analyze-image) (Shen, 2021) to be in the path.

## Methods

### Input layout specifications

The input for each function in CBMAT is a .txt file containing metadata and foci of a set of experiments. The file has to strictly adhere to the following specifications:

- Each line containing metadata must begin with **//.**
- For each experiment, the first line of metadata must contain information of the first author and year of publication, in the format **author,yyyy:**. Possible further details appearing after **:** are not considered by the functions in CBMAT and are therefore not needed (but can be present).
- For each experiment, the last line of metadata must contain the number of subjects included in the neuroimaging experiment, in the format **Subjects=xx.**
- x,y,z coordinates for each focus must be listed on a dedicated line, with space as separator, and **.** as decimal separator.
- An empty line must be left between the last focus of one experiment and the metadata of the following one.
- No header can be included in the file (i.e., the first line of the document must contain the metadata of the first experiment in the format described above).

**Fig. 1:**
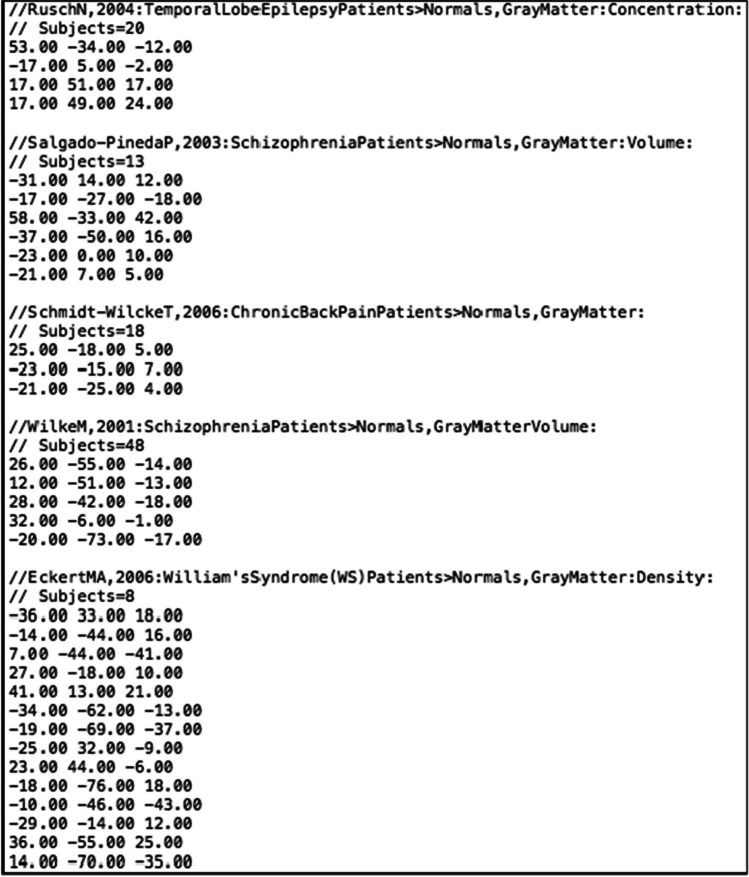
The first lines of an input .txt file showing the required layout

An example of an input .txt file is shown in Fig. 1.

The layout detailed above follows the standard for files created with Sleuth software (http://brainmap.org/sleuth/), with the exception that the first line specifies the reference standard space. The output .txt file of each function in CBMAT follows the same layout, so that it can always be correctly processed with further functions from the toolbox.

### Preparatory procedures on the dataset

*load_foci(foci)*

**load_foci** is the function which allows one to load the .txt data into MATLAB®. The output **Z** is an N×2 cell containing both foci coordinates and metadata of the experiments originally included in the input file, after exclusion of possible duplicates. To be treated as a duplicate, an experiment has to report identical metadata (including the same number of subjects) and identical foci (i.e., same set of x,y,z coordinates) of another experiment in the dataset. **load_foci** is internally called at the beginning of each of the other functions included in CBMAT, but it is also provided as a separate function.

#### Removing non-independent within-study experiments

*remove_multiple(foci,mode,seed)*

One of the central tenets behind the computation of a CBMA is that experiments included are assumed to rely on independent samples from the population of interest (Fox et al., 2014; Müller et al., 2018; Tahmasian et al., 2019). Therefore, the same group of subjects should have been analyzed in no more than one of the considered experiments (Liloia et al., 2021; Liloia, Cauda, et al., 2022a; Liloia, Crocetta, et al., 2022b) to prevent spurious results related to overlapping population within the same paper (i.e. selection bias) (Hutton and Williamson, 2000; Williamson et al., 2005). To overcome this potential bias, **remove_multiple** can be used to sample only one experiment among those which came from the same paper, identified on the basis of first author and year of publication information. When the argument **mode** is set to 1, the experiment with the greater sample size is retained. In the case of more than one experiment with the same sample size, one of them is chosen at random. When the argument **mode** is set to 2, the experiment to be retained is selected at random. The method used is reflected in the name assigned to the output .txt file. Note that in both cases, the behavior of the random number generator process can be controlled for reproducibility purposes by assigning an integer value to the optional argument **seed**. The variable **p** returns the number of experiments still included in the dataset after the procedure. Of note, different criteria could be determined for selection, such as the diagnosis of the clinical group or the functional magnetic resonance imaging (fMRI) task used. Since these are highly variable depending on the research hypothesis, it was not possible to implement them in an automated and standardized way. Nevertheless, **remove_multiple** could be used even after a first manual screening. If the researcher is aware that two or more experiments coming from the same paper are indeed based on independent samples, a letter can be added immediately after the year of publication in the input .txt file to avoid erroneous rejection. This means that two experiments both originally appearing as ‘//AndersonT,2023:’ will be modified as ‘//AndersonT,2023a:’ and ‘//AndersonT,2023b:’. The following command is an example of using **remove_multiple** with the random mode and the value 10 to initialize the random number generator:

$$>>\textrm{p}=\textrm{remove}\_\textrm{multiple}\left({}^{\prime}\textrm{test}\_\textrm{foci}.{\textrm{txt}}^{\prime },2,10\right);$$

#### Tissue selection

*filter_by_tissue(foci,ROI)*

Usually, CBMAs are computed on the whole brain (Eickhoff et al., 2012). However, for some specific research questions it might be preferable to limit the investigation to gray matter (GM) or white matter (WM) only. **filter_by_tissue** allows one to retain in each experiment only foci located inside the tissue of interest. This is achieved by specifying the argument **ROI**, a .nii binary mask containing GM or WM voxels only. Notably, any user-defined segmented mask is accepted, provided it is in Talairach standard space with 2×2×2 mm resolution. The input .txt file for **filter_by_tissue** must contain stereotactic coordinates in Talairach standard space as well. The variable **p** returns the number of experiments still included in the dataset after the procedure. In fact, if an experiment has no foci in the desired tissue, it is no longer included in the output file **foci_tissue.txt**. In the case of an input file with coordinates in MNI space, these can be converted to Talairach space using the external resource **icbm2tal** (Lancaster et al., 2007). The same tool can also be used to transform the output of **filter_by_tissue** into MNI space. The following command is an example of using **filter_by_tissue**:

$$>>\textrm{p}=\textrm{filter}\_\textrm{by}\_\textrm{tissue}\left({}^{\prime}\textrm{test}\_\textrm{foci}.{\textrm{txt}}^{\prime },{}^{\prime}\textrm{GM}\_\textrm{mask}.{\textrm{nii}}^{\prime}\right);$$

Of note, the software Sleuth has an option to specify the tissue during the search procedure. The advantage of using **filter_by_tissue** is that it can also process experiments not included in the BrainMap database.

#### Preparing data for MACM

*prepare_MACM(foci,ROI)*

As described in the introduction, CBMAs return a whole-brain pattern of effect meta-analytically associated with a given cognitive domain or brain disorder. However, the straightforward inference of connectivity pathways from CBMA maps should be undertaken with caution. In fact, the conjoint involvement of brain regions A, B and C in a pattern does not reveal the exact relationship between them. For example, C could be directly connected with B but not with A. A more correct approach in these cases is meta-analytic connectivity modeling (MACM) (Robinson et al., 2010). Briefly, this method allows one to compute a meta-analysis considering only experiments that reported at least one focus of effect in a given region of interest (ROI), thus ensuring that every other peak of effect found in the brain has a direct connection with the selected ROI (for an implementation of MACM in the context of brain disease see Nani et al., 2021, and Manuello, Mancuso, et al., 2022b). Manually preparing data to compute MACM is particularly hard, as it implies checking the foci locations one by one. **prepare_MACM** implements this procedure automatically, considering the user-defined .nii ROI specified through the argument **ROI**. The .nii file must be in Talairach standard space with 2×2×2 mm resolution, and the same space must be used for the stereotactic coordinates in the input .txt file as well. In case of a non-binary ROI, it will be automatically binarized using 0 as a threshold. Any conversion of foci coordinates between standard spaces can be performed as explained above. The variable **p** returns the number of experiments still included in the dataset after the procedure. In fact, if an experiment has no foci in the specified ROI, it is no longer included in the output file **foci_MACM.txt**. The following command is an example of using **prepare_MACM**:

$$>>\textrm{p}=\textrm{prepare}\_\textrm{MACM}\left({}^{\prime}\textrm{test}\_\textrm{foci}.{\textrm{txt}}^{\prime },{}^{\prime}\textrm{ROI}.{\textrm{nii}}^{\prime}\right);$$

Of note, the software Sleuth has an option to specify an ROI during the search procedure. The advantage of using **prepare_MACM** is that it can also process experiments not included in the BrainMap database.

### Preparatory procedures for post hoc analyses

One of the main elements of concern in CBMAs is the possibility of the presence of one experiment (or a few experiments) driving the results (Eickhoff et al., 2016). While the standard output of GingerALE provides diagnostic information in this sense, further ad hoc validation procedures can be implemented and prepared with CBMAT. Having said this, it must be mentioned that there is currently no gold standard or best practices for evaluating the results of post hoc evaluation in CBMAs. Therefore, any interpretation should be made with caution. Although the focus of this work is on how to prepare data for subsequent analyses rather than on the analyses themselves or the evaluation of the obtained results, we propose a very intuitive and straightforward approach based on the voxel-wise frequency of appearance of the effect. In other words, as the strategies described below are essentially based on the repetition of the CBMA analysis on different partitions of the original dataset, it is then possible to determine for each voxel the number of tested conditions in which a significant effect is found. This results in a robustness index, where greater values mean stronger generalizability and replicability across subsamples.

#### Preparing data for LOEO

*create_LOEO(foci,add)*

The leave-one-out approach is widely applied in machine learning as a cross-validation scheme. Its extension to CBMAs was recently proposed to control the effect of each single experiment on the results obtained for the complete dataset (Fu et al., 2021). Starting from a .txt input file with *n* experiments in it, **create_LOEO** generates *n* output .txt files each containing *n* – 1 experiments. At every iteration, the excluded experiment changes, so that all *n* output files generated differ from each other, and each of the *n* experiments is excluded once. A different behavior can be obtained setting the argument **add** to 1. In this case, the procedure becomes incremental, so that at each of the *n* – 1 iterations, one more experiment is excluded, following the order of appearance in the input .txt file. The variable **p** returns the number of subsamples generated. The following command is an example of using **create_LOEO** with the incremental option:

$$>>\textrm{p}=\textrm{create}\_\textrm{LOEO}\left({}^{\prime}\textrm{test}\_\textrm{foci}.{\textrm{txt}}^{\prime },1\right);$$

#### Preparing data for subset analysis

*create_subsets(foci,ns,nexp,rep)*

An alternative approach to validation in CBMAs is to repeat the analysis on several subsamples of the main dataset. The most common implementation is split-half analysis, in which each half of the dataset is analyzed separately, or the boot-strap analyses, in which several subsamples are iteratively generated and analyzed. Both solutions can be obtained with **create_subsets**, depending on the value assigned to the arguments. Specifically, the argument **ns** sets the number of subsamples to be created, while **nexp** specifies the size of each subsample. The argument **rep** controls replacement: when set to 1, an experiment can be included in more than one subsample, while a value of 0 allows each experiment to be included in only one subsample. When **rep** is set to 0, **nexp** is automatically adjusted to create **ns** subsamples of equal size. In this case, if **nexp** is not a divisor of the total number of experiments in the input .txt file, an extra subsample is created containing the remaining experiments. The variable **p** returns the number of subsamples generated. The following command is an example of using **create_subsets** to prepare a split-half analysis from an input file with 10 experiments (i.e., two subsamples with five different experiments each, no replacement allowed):

$$>>\textrm{p}=\textrm{create}\_\textrm{subsets}\left({}^{\prime}\textrm{test}\_\textrm{foci}.{\textrm{txt}}^{\prime },2,5,0\right);$$

From the same file of 10 experiments, this alternative command can be used to prepare a boot-strap analysis (i.e., four subsamples with five different experiments each, replacement allowed):

### Dataset evaluation

*dataset_hist(foci,sbins)*

In addition to the above-described functions for data preparation, CBMAT also includes the graphical function **dataset_hist** to visualize relevant features of the dataset under analysis, namely the distribution of the sample sizes of the experiments included, and their year of publication. The former is a key factor when performing CBMAs through ALE, as this algorithm also models the reliability of each experiment on the basis of its sample size (Eickhoff et al., 2016). As a general rule, a distribution highlighting a majority of experiments with a sample size smaller than 10 subjects should be a warning regarding the reliability of the obtainable results (Liloia et al., 2021; Müller et al., 2018). Moreover, based on the distribution, it is possible to determine a cutoff value on the sample size for the experiments to be further included for subsequent analyses. All these aspects can be verified through the output image **sample_size.jpg**, showing a set of histograms together with a reference line corresponding to the mean sample size across the dataset. The argument **sbins** allows one to specify the number of bins to be shown in the central histogram. The histogram on the top is built with a number of bins automatically defined by MATLAB, while the bottom one shows as many bins as the different values of sample size present in the dataset. The aim of this figure is to provide coarse and very detailed limits to guide the selection of an optimal number of bins to be used to draw inference form the histogram. The distribution of the year of publication can be used as a diagnostic for two aspects: first, it helps to verify whether the dataset is updated, which is a strict prerequisite for every kind of meta-analysis. Second, it can help to identify experiments published before the introduction of a diagnostic update or methodological and conceptual innovation particularly relevant for the field under investigation, which could therefore act as outliers. The output image **year_of_publication.jpg** shows the histogram together with a reference line corresponding to the median of the distribution across the dataset. Note that in this case, the number of bins cannot be defined by the user. Both the average sample size and the median year of publication are stored in the variables **m_subj** and **m_year,** respectively**.** Of note, **dataset_hist** can be used as diagnostic on the output of any other function in CBMAT. The following command is an example of using **dataset_hist** with automatic estimate of the number of bins﻿:

$$>>\left[\textrm{m}\_\textrm{subj},\textrm{m}\_\textrm{year}\right]=\textrm{dataset}\_\textrm{hist}\left({}^{\prime}\textrm{test}\_\textrm{foci}.{\textrm{txt}}^{\prime },0\right);$$

## Implementation

We now show an example of the functionalities of CMBAT through a complete run on a test dataset including 34 experiments (see Fig. 1 for a visualization of the first lines of the input .txt file). As explained above, functions are designed to allow a combined use, but they can also each be used separately depending on the researcher needs. We are therefore not recommending that this specific order of analyses has to be followed for correct usage of CBMAT.

First, we use **dataset_hist** to inspect the original dataset.

$$>>\left[\textrm{m}\_\textrm{subj},\textrm{m}\_\textrm{year}\right]=\textrm{dataset}\_\textrm{hist}\left({}^{\prime}\textrm{test}\_\textrm{foci}.{\textrm{txt}}^{\prime },15\right);$$

**Fig. 2:**
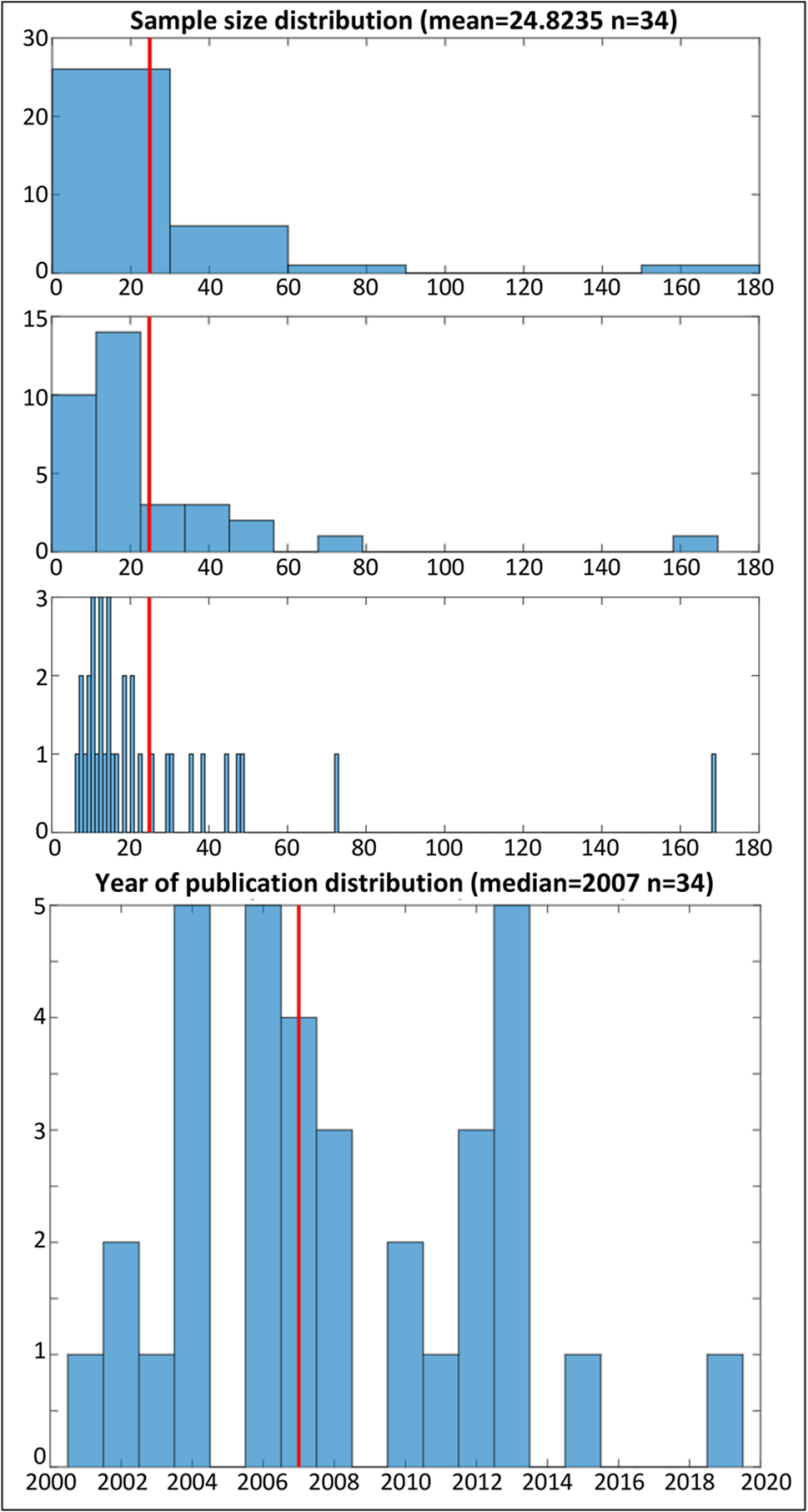
The output of dataset_hist function referring to the file test_foci.txt for sample size (top) and year of publication (bottom). The red line in the top three histograms marks the mean sample size computed across the dataset. The red line in the bottom histogram marks the median year of publication across the dataset. In the second histogram from the top, the number of bins was set to 15 based on the value selected for the argument **sbins**.

The output confirms that 34 experiments were included (Fig. 2).

The mean sample size is 25, and most of the experiments have around 20 subjects. The median year of publication is 2007, and the distribution suggests that the dataset is quite updated.

As a second step, we use **remove_multiple** to exclude potential multiple experiments from the same paper. The selection is made in random mode without specifying a value for the **seed** argument to control the random sampling behavior.

$$>>\textrm{p}=\textrm{remove}\_\textrm{multiple}\left({}^{\prime}\textrm{test}\_\textrm{foci}.{\textrm{txt}}^{\prime },2\right);$$

**Fig. 3:**
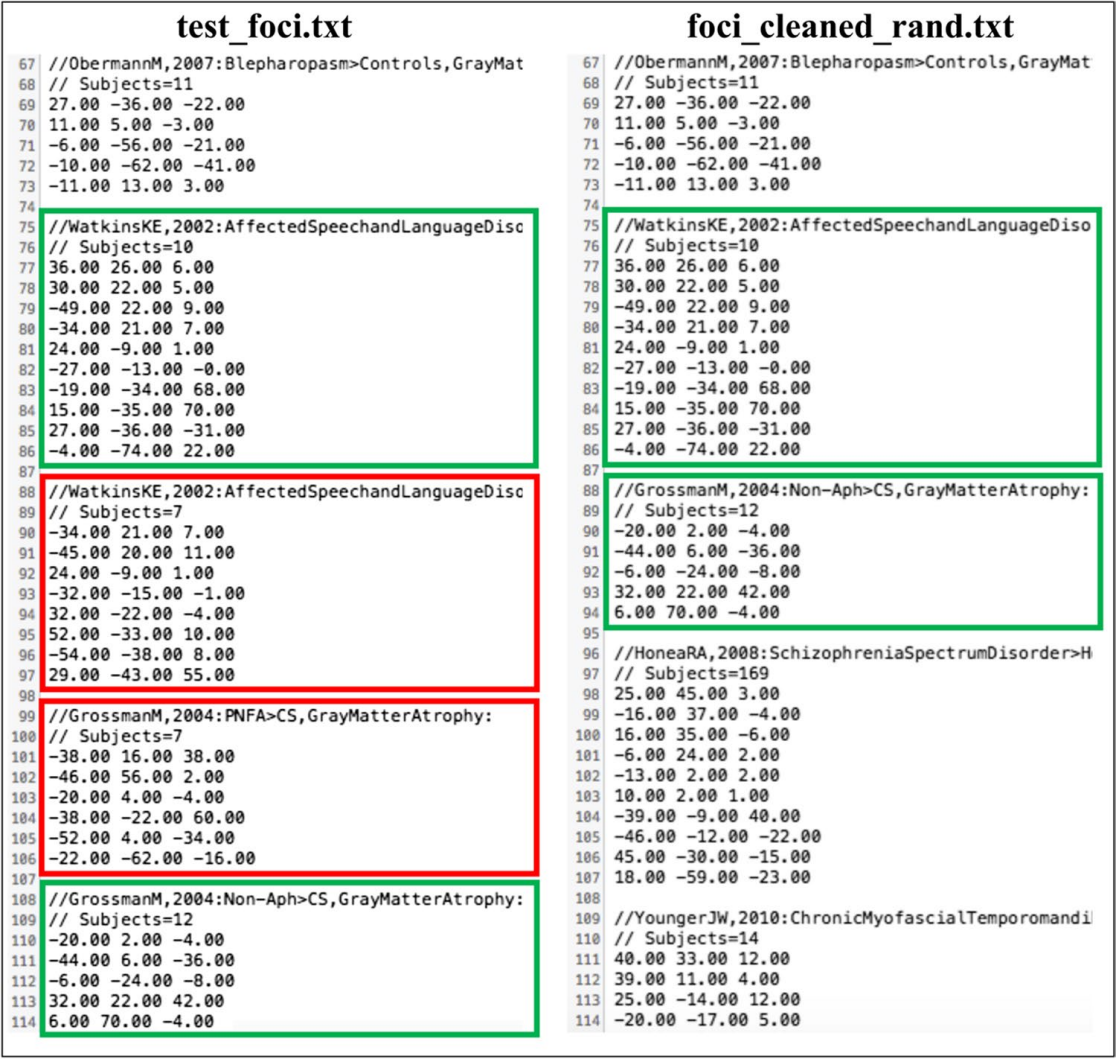
A comparison of **test_foci.txt** (left) and **foci_cleaned_rand.txt** (right) obtained through **remove_multiple** function. The experiments that were retained are marked in green in both files, while the discarded experiments are marked in red in **test_foci.txt** only

The variable **p** informs us that 30 experiments are now included in the file **foci_cleaned_rand.txt**, and four experiments have therefore been discarded from the dataset (Fig. 3).

Third, we use **filter_by_tissue** to retain only foci located in the GM.

$$>>\textrm{p}=\textrm{filter}\_\textrm{by}\_\textrm{tissue}\left({}^{\prime}\textrm{foci}\_\textrm{cleaned}\_\operatorname{rand}.{\textrm{txt}}^{\prime },{}^{\prime}\textrm{GM}\_\textrm{mask}.{\textrm{nii}}^{\prime}\right);$$

**Fig. 4:**
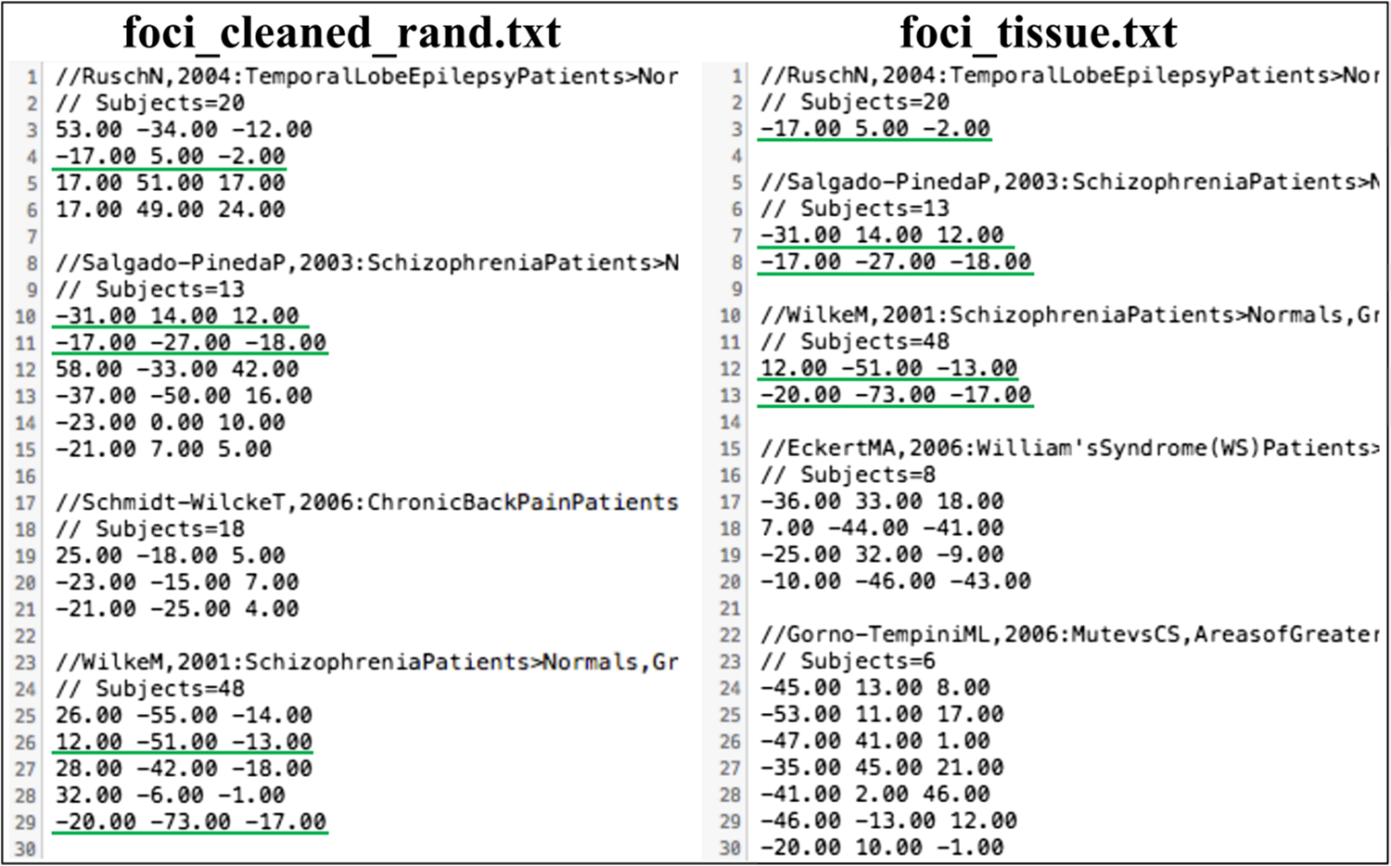
A comparison of **foci_cleaned_rand.txt** (left) and **foci_tissue.txt** (right) obtained through the **filter_by_tissue** function. The foci that were retained are marked in green in both files. It can be seen that the entire experiment by Schmidt-WilckeT, 2006 is no longer present in **foci_tissue.txt**, as it did not include foci located in the GM

The variable **p** informs us that 23 experiments now remain in the file **foci_tissue.txt**, as seven discarded experiments had no foci localized in the GM (Fig. 4).

Fourth, we use **prepare_MACM** to select experiments with at least one focus localized in a user-defined ROI of the lentiform nucleus.

$$>>\textrm{p}=\textrm{prepare}\_\textrm{MACM}\left({}^{\prime}\textrm{foci}\_\textrm{tissue}.{\textrm{txt}}^{\prime },{}^{\prime}\textrm{ROI}.{\textrm{nii}}^{\prime}\right);$$

**Fig. 5:**
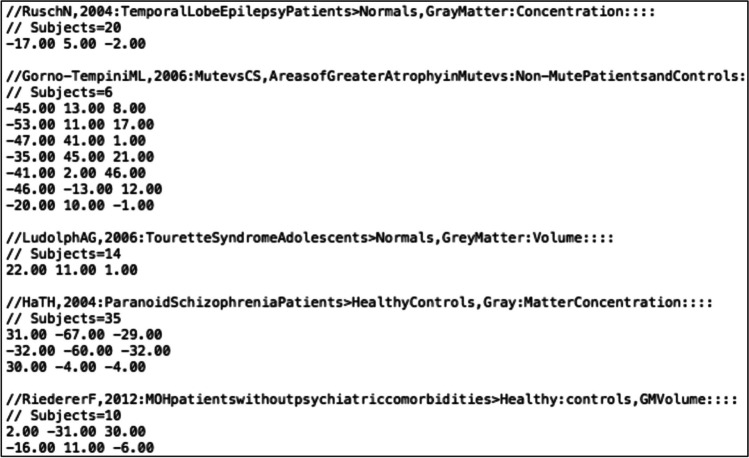
The **file foci_MACM.txt** obtained through the function **prepare_MACM**

The variable **p** informs us that five experiments are now included in the file **foci_MACM.txt** (Fig. 5).

As this complete preprocessing led to a very small test dataset, post hoc analyses were performed on the file **foci_cleaned_rand.txt**, assuming therefore a standard CBMA on both GM and WM, rather than a MACM on GM only, as the main analysis.

First, we use **create_LOEO** to prepare data for a standard LOEO analysis.

$$>>\textrm{p}=\textrm{create}\_\textrm{LOEO}\left({}^{\prime}\textrm{foci}\_\textrm{cleaned}\_\operatorname{rand}.{\textrm{txt}}^{\prime },0\right);$$

**Fig. 6:**
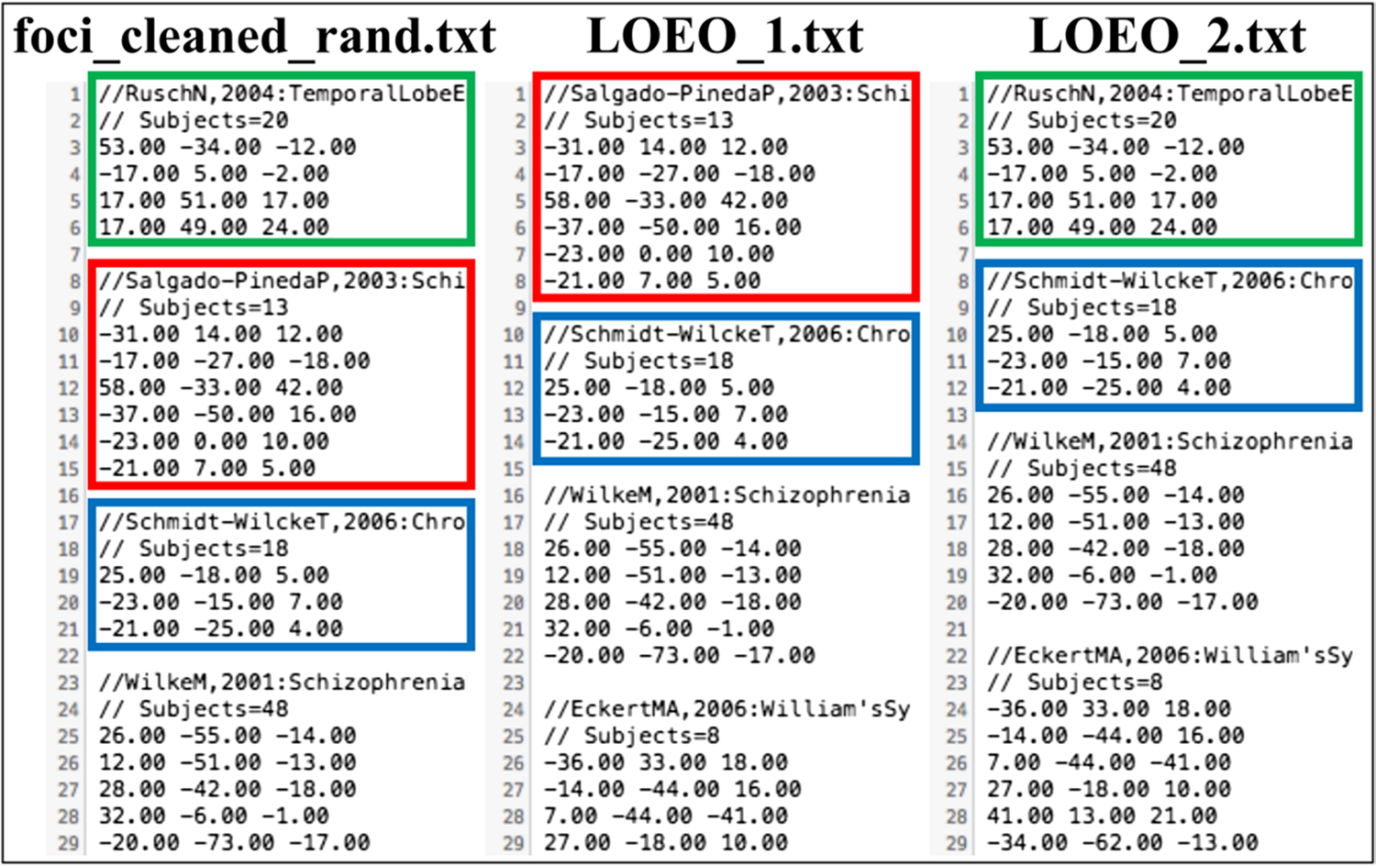
A comparison of **foci_cleaned_rand.txt** (left), **LOEO_1.txt** (center) and **LOEO_2.txt** (right), both obtained through the **create_LOEO** function. This shows that in **LOEO_1.tx**t it was the first experiment (green boxes) to be removed, while in **LOEO_2.tx**t it was the second one (red boxes)

The variable **p** informs us that 30 subsamples were created and saved in the files **LOEO_1.txt**, **LOEO_2.txt**, …, **LOEO_30.txt** (Fig. 6).

Finally, we use **create_subsets** to prepare data for a split-half analysis.

$$>>\textrm{p}=\textrm{create}\_\textrm{subsets}\left({}^{\prime}\textrm{foci}\_\textrm{cleaned}\_\operatorname{rand}.{\textrm{txt}}^{\prime },\textrm{2,15,0}\right);$$

The variable **p** informs us that two subsamples were created and saved in the files **sub_sample_1.txt** and **sub_sample_2.txt**.

We can now again use **dataset_hist** to inspect the two obtained subsamples.

$${\displaystyle \begin{array}{c}>>\left[\textrm{m}\_\textrm{subj},\textrm{m}\_\textrm{year}\right]=\textrm{dataset}\_\textrm{hist}\left({}^{\prime}\textrm{sub}\_\textrm{sample}\_1.{\textrm{txt}}^{\prime },20\right);\\ {}>>\left[\textrm{m}\_\textrm{subj},\textrm{m}\_\textrm{year}\right]=\textrm{dataset}\_\textrm{hist}\left({}^{\prime}\textrm{sub}\_\textrm{sample}\_2.{\textrm{txt}}^{\prime },20\right);\end{array}}$$

**Fig. 7:**
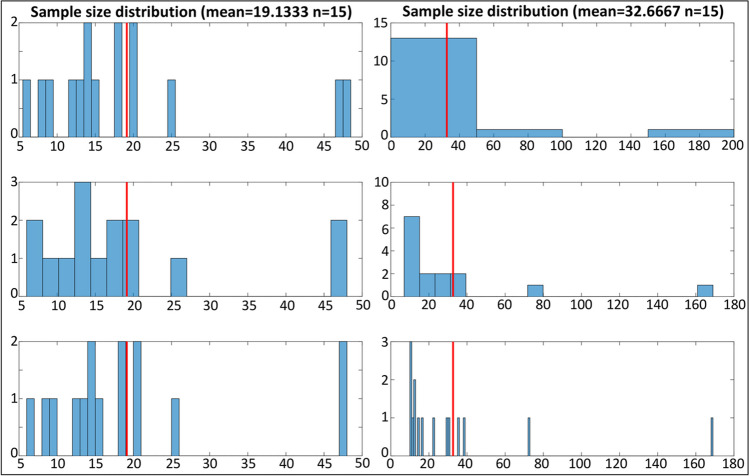
A comparison of the sample size distribution histograms obtained through the **dataset_hist** function for the files **sub_sample_1.txt** (left) and **sub_sample_2.txt** (right). The red lines mark the mean sample size computed across each subsample

The histograms of the sample size distributions (Fig. 7) confirm that they each contain 15 experiments, as specified in **create_subsets**. It can also be observed that the mean sample size varies greatly between the two subsamples, mostly due to the inclusion of an experiment with around 160 subjects in subsample 2. In similar cases, bootstrapping the split-half procedure is often preferred in order to balance the sampling. This can be easily obtained through multiple iterations of **create_subsets**, as it implements a random selection process for the experiments.

## Conclusions

CBMAT is a MATLAB® toolbox developed to assist researchers during data preparation and post hoc analysis in the field of CBMA. Traditionally, all these procedures have had to be manually performed, making the process highly time-consuming and prone to error. CBMAT now allows them to be performed in a fast and automated manner. Moreover, it can be flexibly adapted to the methodological needs of a specific investigation, thanks to the integrated but independent implementation of each function. This toolbox contributes, therefore, to boosting the field of CBMA, helping researchers simplify and improve their processing routines.

## Data Availability

The dataset used for the example run described here can be downloaded from https://github.com/Jordi-Manuello/CBMAT.git. The same data can be also freely obtained from the BrainMap database.

## Code availability

The whole MATLAB code of CBMAT can be downloaded from https://github.com/Jordi-Manuello/CBMAT.git.
